# Smaller cross-sectional areas of the hamstring tendon measured from preoperative ultrasonography are likely to need additional gracilis harvesting for double-bundle anterior cruciate ligament reconstructions

**DOI:** 10.1186/s43019-020-00052-5

**Published:** 2020-07-08

**Authors:** Kazumi Goto, Masahiko Hara, Yoshiyuki Yamazaki, Taihei Urata, Yuki Shimizu, Naofumi Shimizu

**Affiliations:** 1 Department of Orthopaedic Surgery, Toshiba Rinkan Hospital, Toshiba Rinkan Hospital, 7-9-1, Kamitsuruma, Minami-ku, Sagamihara-Shi, Kanagawa 252-0385 Japan; 2 Japan Society of Clinical Research, Chuoh-ku, Tokyo, Japan

**Keywords:** Double-bundle anterior cruciate ligament reconstruction, Semitendinosus tendon, Graft choice, Gracilis tendon, Ultrasonography

## Abstract

**Background/Purpose:**

Hamstring tendon autografts are commonly used for double-bundle anterior cruciate ligament reconstruction (DB-ACLR). If the volume of the semitendinosus (ST) tendon is insufficient, the gracilis (G) tendon is also harvested. Additional harvesting of the G autograft can affect patients’ short-term postoperative outcome, such as muscle recovery; thus, preoperative information about whether an additional G autograft is needed would be useful. The purpose of this study was to investigate whether preoperative measurement of the ST tendon using ultrasonography could inform the intraoperative decision to harvest the G tendon.

**Methods:**

We enrolled 20 patients (13 men and seven women) who underwent DB-ACLR between October 2017 and March 2019. The mean patient age was 28.5 years. The ipsilateral ST tendon was measured using ultrasonography before surgery. Measurements included the diameter and breadth of the short-axis image. The cross-sectional area (CSA) was calculated from these measurements. During surgery, when two grafts with diameters of ≥ 5.0 mm could not be made, the G tendon was also harvested. Patients were categorized into two groups: the ST group where only the ST tendon was harvested, and the semitendinosus gracilis tendon (STG) group where the ST and G tendons were both harvested. The CSA value was compared between the two groups, and the cutoff value was calculated.

**Results:**

In the ST group (*n* = 8), the mean diameter and breadth of the semitendinosus tendon were 4.21 and 2.34 mm, respectively. In the STG group (*n* = 12), the mean diameter and breadth of the ST tendon were 3.39 and 1.78 mm, respectively. The CSAs calculated for the ST group and the STG group were 7.74 mm^2^ and 4.79 mm^2^, respectively. A cutoff value of 7.0 mm^2^ was found to correspond to a specificity and sensitivity to harvest the G tendon of 87.5% and 75.0%, respectively.

**Conclusions:**

The preoperative CSA of the ST tendon determined using ultrasonography can, therefore, be informative for deciding whether to harvest the G tendon for DB-ACLR. The results of this study provide valuable information for graft selection in anterior cruciate ligament reconstruction.

**Level of Evidence:**

IV (Retrospective case series design).

## Background/purpose

Anterior cruciate ligament (ACL) injuries are among the most common sports injuries of the knee [1]. The ACL is a double-bundled ligament containing the anteromedial (AM) and posterolateral (PL) bundles, which together provide anterior and rotational stability of the knee [2, 3]. Arthroscopic ACL reconstruction has long been the gold standard treatment for active patients with an ACL injury [4, 5]. Currently, the arthroscopic double-bundle (DB) technique has been suggested as an alternative intervention to the conventional single-bundle (SB) technique—which restores the ACL as only one bundle [6, 7]—for restoration of native anterior and rotational stability.

Hamstring autografts are most commonly used for DB reconstruction [8]. Numerous hamstring preparation techniques have been described, the most common of which include two double-looped semitendinosus (ST) tendon grafts and two double-looped ST tendon with one double-looped gracilis (G) tendon grafts. When the harvested double-looped portion of the ST tendon is thick enough, only the ST tendon is needed for AM- and PL-bundle grafts. On the other hand, when the harvested double-looped portion of the ST tendon is not thick enough, the G tendon can be harvested and used in addition [9].

Although it is crucial to make grafts of sufficient size, functional loss as a result of harvesting the G tended is a concern. Preservation of the gracilis muscle may reinforce the action of the hamstrings in deep knee flexion and potentially help compensate for the loss of the ST graft, and may increase postoperative hamstring strength [1, 2, 4–18]. Therefore, other graft choices should be considered for athletes who use deep knee flexion for their specific activity, such as a ballet dancers. A preoperative imaging examination must be useful if it can be used to predict whether the G tendon needs to be harvested preoperatively. This idea was our motivation for this study.

Several previous studies have investigated the prediction of graft size using magnetic resonance imaging (MRI) and/or ultrasonography (US) [15, 18–20]. However, these studies focused on the single-bundle ACL reconstruction (SB-ACLR) technique, and few have focused on double-bundle ACL reconstruction (DB-ACLR). Furthermore, studies that used MRI had not obtained “true” axial views because the hamstring tendons tilt on the back side of the thigh. In the present study, the size of the ST tendon was measured using US, which provides a true axial view. Our study is the first to evaluate the measurement of the ST tendon by using US in DB-ACLR.

The purpose of this study was to investigate whether the preoperative measurement of the ST tendon using US would help with the intraoperative decision to harvest the G tendon. The hypothesis of this study was that the patients who had a larger cross-sectional area (CSA) of the ST tendon measured preoperatively would not require an additional G tendon to be harvested for DB-ACLR.

## Methods

### Subjects

This study used a diagnostic, retrospective case series design (Level-IV Evidence), and was approved by the Institutional Review Board. Written informed consent was obtained from each patient for publication of this case series and the accompanying images.

Twenty patients (13 men and seven women, mean age at the time of surgery 28.5 ± 10.2 years) who underwent DB-ACLR using hamstring autografts between October 2017 and March 2019 were included in this study. The inclusion criteria were as follows: primary ACL reconstruction, using an ipsilateral hamstring tendon autograft, and ST tendon of the need properly confirmed by preoperative US. The exclusion criteria were as follows: a history of hamstring trauma, a history of revision ACL reconstruction, a history of trauma around the knee, multiple ligamentous injuries, and previous septic arthritis. All surgeries were primary surgeries conducted by the same orthopedic surgeon (KG). All data were collected and analyzed retrospectively from an Institutional-Review-Board-approved database, and the study was carried out according to the principles of the Declaration of Helsinki.

### Ultrasonographic evaluation

**Fig. 1:**
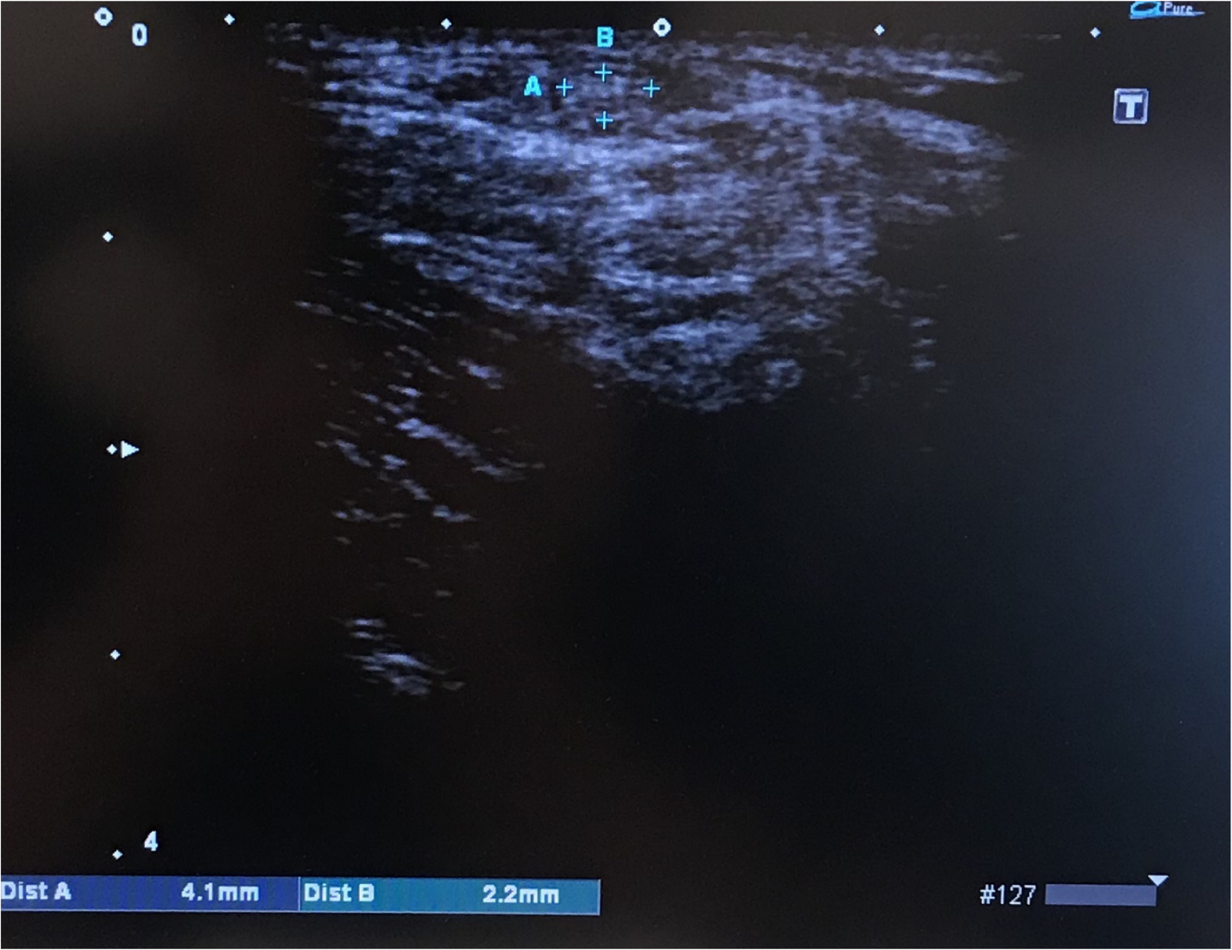
Ultrasonography images of the short-axis view. The diameter (**a**) and breadth (**b**) of the semitendinosus tendon

All measurements of ST tendons using US were performed 1 day before surgery by a single surgeon (KG), who has extensive experience with musculoskeletal US. The US examinations were performed using a Viamo SSA-640A (Toshiba, Tokyo, Japan) with a 7.5-MHz linear probe (PLT-704AT, Toshiba, Tokyo, Japan) for all measurements. The patients were positioned in the prone position. The ST was identified with the knee extended after repeated scans and palpations. The crease level behind the knee was defined as the measurement zone. Tendons were measured using the true axial view, which showed the tendon to be most narrow in the transverse US image (Fig. 1a, b). Measurements included the diameter and breadth, which were used to calculate the elliptical CSA. These measurements and calculations were performed twice by one author (KG), and the mean CSA calculated from these results. In addition, to evaluate intraobserver and interobserver reproducibilities, the measurements were performed three times by one examiner (KG) and once by two examiners (TK, RT) on eight knees randomly selected from the volunteer staff in our institution. The intraclass and interclass correlation coefficients for the diameter were 0.95 and 0.81, respectively.

### Surgical techniques

All operations were performed arthroscopically with tourniquet application. All hamstring tendon harvesting was performed by one surgeon (KG) who routinely performs hamstring autograft ACL reconstructions. The ST tendon was harvested by first creating a short, oblique 3-cm incision on the pes anserinus of the injured leg. An incision in the sartorial fascial layer exposed the ST and G tendons. The ST tendon was released distally and stitched using a baseball-suture technique. The ST tendon was harvested using a closed tendon stripper, and all the muscle fibers were removed.

The harvested ST tendon was attached to the graft preparation station (Graftmaster, AcufexTM, Smith & Nephew, Memphis, TN, USA) to form two 6–7-cm double-looped grafts. This procedure was performed by the other authors (YY, UT, and SY). The aim of the graft was to ensure that each socket of the AM and PL tunnels in both the femur and tibia was more than 12 mm. The graft passed through an open-hole sizing block (of 0.5 mm incremental diameter from 4.5 mm to 11 mm) to measure its diameter. The G tendon was also harvested in cases where it was not possible to make two grafts with diameters of ≥ 5.0 mm from the ST tendon. The decision to harvest the G tendon was made by the surgeons who were preparing the graft (YY, TU, and YS).

A far antero-medial portal was routinely created as an additional third portal for drilling both AM and PL tunnels of the femoral bone. Femoral-graft fixation was achieved using the EndoButton CL (Smith & Nephew, Memphis, TN, USA). Tibial-graft fixation was achieved using the screw post-method fixation with a cortical screw.

### Statistical analyses

Patients were categorized into two groups as follows: the ST group, in whom only the ST tendon was harvested; and the semitendinosus gracilis tendon (STG) group, in whom the G tendon was also harvested. To evaluate between-group differences, Fisher’s exact test was used to compare nominal variables (gender and side). The Mann-Whitney *U* test was used to compare continuous variables (age, height, body weight, diameter, breadth, and CSA). Receiver operating characteristic (ROC) analysis was performed to determine the area under the ROC curve (AUC), which demonstrates the diagnostic accuracy of a test ranging from 0.5 to 1.0. The generated ROC curves were used to determine a possible cutoff value for preoperative assessment of whether to harvest the G tendon. The cutoff value was referred to as the minimum value required not to harvest the G tendon. A *p* value of < 0.05 was considered to be statistically significant. All statistical analyses were performed using R software packages (version 3.2.1; R Development Core Team). A minimum sample size of 16 patients in each cohort was required to provide appropriate power (beta 1/4 0.80) by using a significance level of 0.05 because of a clinically significant CSA difference of 3 mm^2^, as calculated in previous studies [16, 20].

## Results

Demographic data are presented in Table 1. The STG group comprised eight cases while the ST group included 12. The dimensions of the harvested tendons and corresponding mean CSAs are described in Table 2; all dimensions including the CSA were significantly greater in the ST group than in the STG group.

**Table 1:** Demographic characteristics of the study cohort

	ST group (*n* = 12)	STG group (*n* = 8)	*p* value
Sex (M/F)	10/2	3/5	0.0623
Age (years)	29.6 ± 10.7	26.8 ± 10.6	0.417
Side (R/L)	3/9	7/1	0.0198
Height (cm)	172.9 ± 5.8	163.6 ± 6.3	< 0.01
Body weight (kg)	72.9 ± 9.9	60.5 ± 15.2	0.0167

Categorical data are presented as number, continuous data are presented as mean ± standard deviation

*STG* semitendinosus gracilis tendon

**Table 2:** Comparison of the tendon diameter, breath, and cross-sectional area

	Group ST	Group STG	*p* value
Diameter	4.21 ± 0.38	3.39 ± 0.68	0.00329
Breadth	2.34 ± 0.43	1.78 ± 0.42	0.0149
CSA	7.74 ± 1.41	4.79 ± 1.59	0.00296

Continuous data are presented as mean ± standard deviation

*Abbreviations*: *ST* semitendinosus tendon, *STG* semitendinosus gracilis tendon, *CSA* cross-sectional area

**Fig. 2:**
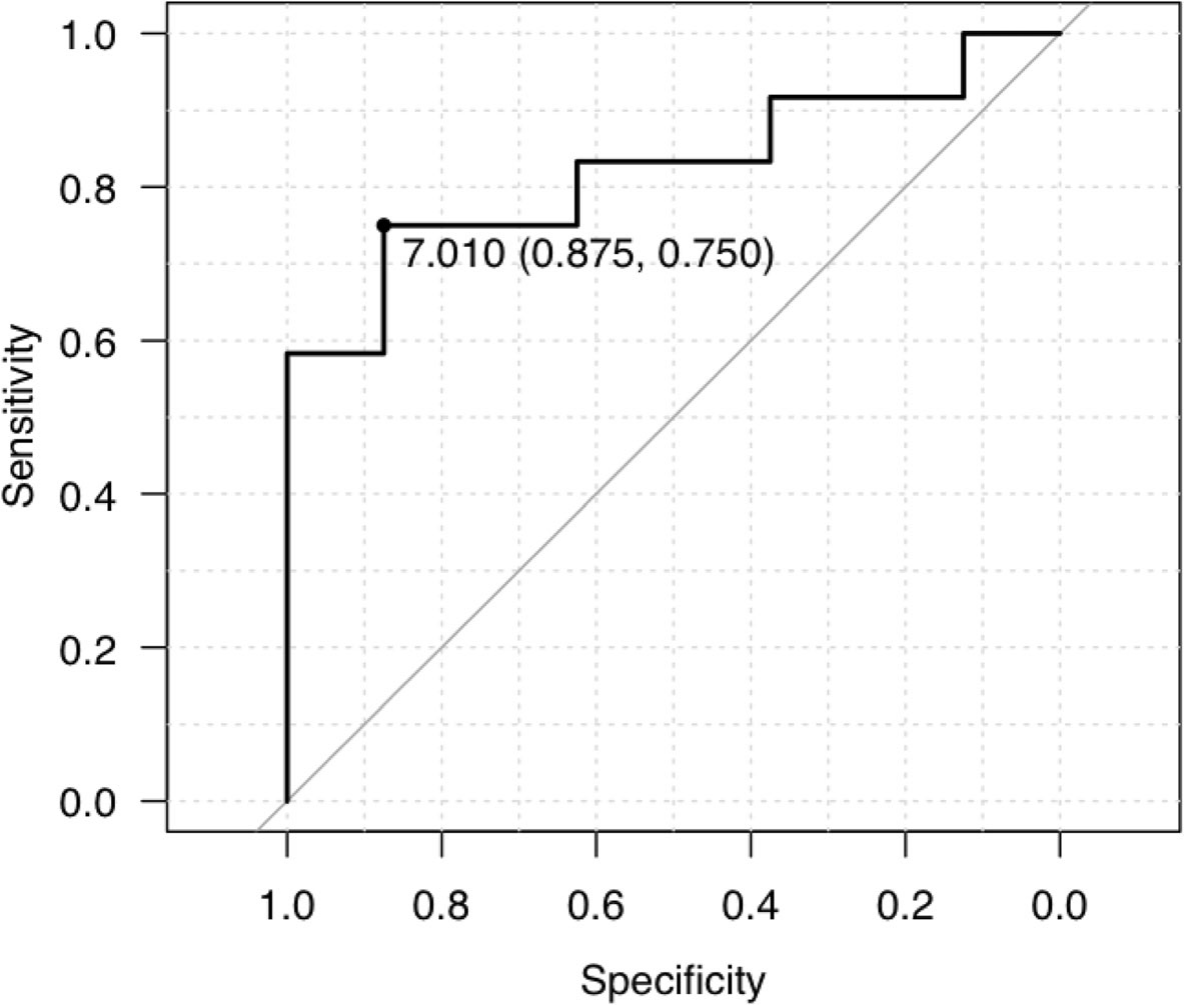
Receiver operating characteristics’ curve analysis. Area under the receiver operating characteristic curve was measured to be 0.823. A cutoff of 7.010, sensitivity of 0.750, and specificity of 0.875 were calculated

Based on ROC analysis, the AUC was found to be 0.823, and the minimum CSA cutoff for not harvesting the G tendon was determined to be 7.01 mm^2^. This corresponded to a specificity of 87.5% and a sensitivity of 75.0% (Fig. 2).

## Discussion

The most important finding of this study was that the preoperative measurement of ST tendon using US is informative for deciding whether the G tendon needs to be harvested in addition to the ST tendon. The results of this study suggest that it is less likely to be necessary to harvest the G tendon in cases where the CSA of the ST is ≥ 7.0 mm^2^. Our primary hypothesis was that the patients who had a larger CSA of the ST tendon measured would not require an additional G tendon to be harvested for DB-ACLR, and our findings support our hypothesis.

In this study, the goal of graft preparation was to make two grafts ≥ 5 mm, based on previous anatomical studies [21, 22]. Sasaki et al. demonstrated that femoral insertion of the ACL was oval, had a short-axis length of 4.6 mm macroscopically, and a direction insertion of 5.3 mm microscopically [21]. In addition, Siebold et al. reported that the midsubstance of the ACL was flat with a mean width of 9.9 mm, thickness of 3.9 mm, and CSA of 38.7 mm^2^ [22]. Based on these anatomical studies, our surgical strategy was to make two grafts measuring ≥ 5.0 mm in diameter, thereby resulting in a CSA > 39.2 mm^2^. However, it was previously unclear what graft size is sufficient for DB-ACLR; thus, it was also unknown whether this strategy would be appropriate. Previous studies have reported that an autograft using a hamstring with a diameter ≥ 8 mm decreases the risk of revision when performing SB-ACLR [10, 11] However, a clear cutoff for the appropriate graft size has not been established for DB-ACLR.

Our surgical concept was based on the premise that the G tendon should be preserved where possible. There have been several studies investigating the advantage of preserving the G tendon after ACLR. In a systematic review assessing whether harvesting of the ST and G tendons leads to postoperative deficits in hamstring strength compared with harvesting of the ST tendon alone, Arden et al. [12] found no differences in isokinetic hamstring strength. Nevertheless, significant hamstring deficits have been observed in isometric-strength testing at 70° and 90° knee-flexion angles in the prone position at 18 months postoperatively [23]. Nakamura et al. [17] reported that the standing knee-flexion angle at 2 years postoperatively was significantly lower in cases where both tendons were harvested. A recent review by Sharma et al. [24] in 2016 reported that harvesting the G tendon caused a significant decrease in hamstring strength in isokinetic testing at 60°/s and isometric-strength testing at 90° of flexion; their follow-up ranged from 6 months to 3 years. However, the overall differences found were in the 3.85–5.55% range, and are not likely to be clinically significant. At high flexion angles (105–110°), a larger difference of 13.68% was found, which likely approaches clinical significance. Therefore, there might be advantages in preserving the G tendon, especially for some specific athletes who perform at high knee-flexion angles or require standing deep knee flexion. Based on these studies, the surgical strategy to preserve the G tendon where possible are justified. For the above reasons, our study focused on preservation of the G tendon using a preoperative US examination, which is widely available in most clinical situations. This is the most remarkable strength of our study.

There have been several previous reports on preoperative measurement of the hamstring tendon using US and/or MRI for the prediction of graft sizes. Galanis et al. [15] assessed the graft diameter preoperatively using US and MRI in 14 male patients undergoing ACL reconstruction with the hamstring tendon. They reported the mean CSA of the ST tendon to be 13.22 mm^2^ from MRI, 12.81 mm^2^ from US, and 13.00 mm^2^ intraoperatively. The final ACL graft diameter displayed correlation coefficients of 0.813 with the MRI CSA and 0.518 with the US CSA. In our study, the CSA of the ST tendon was two- to three-fold lower than that reported by Hamada et al. [16] (10.1 mm^2^), Erquicia et al. [14] (12.4 mm^2^), and Wernecke et al. [20] (16.5 mm^2^). However, one of the reasons may be because the previous authors may not have delineated axial images of the tendons, as they were not truly perpendicular transversal slices. In addition, US examination procedures were not consistent across studies, especially considering that the sites of linear probe positioning were not reported [14, 15, 19]. Furthermore, the knee position varied among studies from full extension [20] to 30° flexion [15] or 90° flexion [14]. Our approach was to use a true axial view, which showed the ST tendon at its most narrow.

In a cadaveric study of 43 male and 50 female individuals in Austria, the mean CSA of the ST tendon was found to be 11.4 mm^2^, with a mean length of 263.7 mm [13, 16, 25]. The detailed demographic data of their study; for example, race, height, and body weight; were not obviously described. However, differences in the skeleton as a result of race could explain the difference between previous studies and our results. Similar results have been reported in a previous study involving a Chinese population [13]. Hamada et al. [16] also reported a similar measurement of ST CSA (10.1 mm^2^) to our study (7.7 mm^2^) when assessing the hamstring tendons of subjects of Japanese descent. Nevertheless, their study measured the hamstring tendon using MRI and, therefore, their measurements could be larger.

### Limitations

Our study has some limitations that should be acknowledged. First, this is a retrospective study carried out at a single institution. Further studies involving multiple medical centres in Japan and, possibly, other Asian countries, are required. Second, all measurements were performed by only one author. There is a possibility of inter-observer error because the US measurements were dependent on individual skill. Future investigations in which all measurements are performed by more than one author could identify and eliminate any possible inter-observer error. Third, all measurements were performed on the crease behind the knee. Hence, observation points should not be fixed contingent on their physical differences. Previous studies prefer the widest point of the medial femoral epicondyle as a measurement point [15, 19]. However, in our view, observations on the narrowest point of the ST tendon could be more important in accurately predicting the graft size. Fourth, a 7.5-MHz linear probe was used in our study, which could be insufficient to precisely analyze small distances; it is desirable to use high linear probes over 10 MHz to ensure precise analysis of these smaller distances during DB-ACLR. Nevertheless, our study indicates the high versatility of the 7.5-MHz linear probe, which is more likely to be available in clinical settings than a high linear probe. Fifth, no clinical outcome was reported in this study. Therefore, our criterion of the graft choice in terms of whether an additional G tendon should be harvested was not justified. Finally, the sample size was small, meaning that the results may vary from those of other studies with larger sample sizes, especially in terms of inter-observer comparisons. Despite these limitations, our results provide a good reference for the decision of harvesting G tendons, as this study is the first to assess measurements for DB-ACLR. This study, therefore, provides valuable information to inform clinical decision-making regarding graft selection for ACL reconstruction.

## Conclusions

The preoperative CSA of the ST tendon determined by using ultrasonography could provide useful information for deciding whether to harvest the G tendon or not for DB-ACLR.

## Data Availability

The datasets during and/or analyzed during the current study are available from the corresponding author on reasonable request.

## Abbreviations

ACL: Anterior cruciate ligament
ACLR: Anterior cruciate ligament reconstruction
SB: Single bundle
DB: Double bundle
ST: Semitendinosus tendon
STG: Semitendinosus gracilis tendon
AM: Anteromedial
PL: Posterolateral
G: Gracilis
MRI: Magnetic resonance imaging
US: Ultrasonography
SB-ACLR: Single-bundle anterior cruciate ligament reconstruction
DB-ACLR: Double-bundle anterior cruciate ligament reconstruction
CSA: Cross-sectional area
ROC: Receiver operating characteristic
